# Non-alcoholic fatty liver disease (NAFLD) is associated with an increased incidence of chronic kidney disease (CKD)

**DOI:** 10.1186/s40001-023-01114-6

**Published:** 2023-04-17

**Authors:** Christoph Roderburg, Sarah Krieg, Andreas Krieg, Münevver Demir, Tom Luedde, Karel Kostev, Sven H. Loosen

**Affiliations:** 1 Department of Gastroenterology, Hepatology and Infectious Diseases, University Hospital Duesseldorf, Medical Faculty of Heinrich Heine University Duesseldorf, Moorenstraße 5, 40225 Duesseldorf, Germany; 2 Department of Surgery (A), University Hospital Duesseldorf, Medical Faculty of Heinrich Heine University Duesseldorf, 40225 Duesseldorf, Germany; 3 Department of Hepatology and Gastroenterology, Charité University Medicine Berlin, 13353 Berlin, Germany; 4 Epidemiology, IQVIA, 60549 Frankfurt, Germany

**Keywords:** Non-alcoholic fatty liver disease, Chronic kidney disease, Non-alcoholic steatohepatitis, NAFLD, NASH, CKD, Metabolic syndrome, Epidemiology

## Abstract

**Background:**

Non-alcoholic fatty liver disease (NAFLD) is the leading cause of chronic liver disease in the western world. The excess mortality in NAFLD patients is strongly related to extrahepatic comorbidities. Recently, an association between NAFLD and chronic kidney disease (CKD) has been reported in various populations.

**Methods:**

Based on the IQVIA Disease Analyzer database, this retrospective study examined two cohorts from Germany matched for sex, age, index year, annual visit frequency, hypertension, and diabetes, including 92,225 patients with and without NAFLD. The incidence of CKD was assessed as a function of NAFLD using Cox regression models.

**Results:**

A total of 92,225 NAFLD patients as well as 92,225 patients without NAFLD were included into analyses. CKD was diagnosed in 19.1% vs. 11.1% of patients with and without NAFLD within the 10 years observation period (*p* < 0.001). Cox regression confirmed a significant association between NAFLD and CKD with a hazard ratio (HR) of 1.80 (95%CI: 1.73–1.86, *p* < 0.001). Subgroup analyses revealed that this association was most pronounced in the age group of 18 to 50 years (HR: 2.13, 95%CI: 1.91–2.37, *p* < 0.001) and among female NAFLD patients (HR 1.85, 95%CI: 1.76–1.95, *p* < 0.001).

**Conclusions:**

The results of this study confirm a significantly increased risk of developing CKD in a large, real-world cohort of adult NAFLD patients in Germany. Interdisciplinary care of NAFLD patients, which is currently gaining importance worldwide, should be considered to include systematic measures for prevention and/or early detection of CKD with the aim of minimizing long-term renal complications.

**Supplementary Information:**

The online version contains supplementary material available at 10.1186/s40001-023-01114-6.

## Introduction

Non-alcoholic fatty liver disease (NAFLD) is the most common cause of chronic liver disease in the Western world, with an estimated prevalence of approximately 25% [1, 2]. According to a US population-based study of 3869 NAFLD patients and 15,209 controls, the incidence of NAFLD increased fivefold from 62 to 329 per 100,000 person-years between 1997 and 2014, and sevenfold in the 18–39 age group [3]. NAFLD is an umbrella term for a broad spectrum of liver diseases ranging from simple non-inflammatory steatosis with little or no progression (NAFL), to non-alcoholic steatohepatitis (NASH) with inflammatory processes and hepatocyte damage, to its complications, fibrosis, cirrhosis or hepatocellular carcinoma [4, 5]. Patients with NAFLD have been reported to have increased all-cause mortality compared to healthy individuals, primarily due to cardiovascular disease, tumor disease, and progression of liver disease itself [6]. NAFLD is considered a hepatic manifestation of the metabolic syndrome, but can also occur independently. In particular, visceral obesity and type 2 diabetes are associated with the presence of NAFLD. The risk of progression from steatosis to NASH and NASH cirrhosis depends on several factors, including the patient's lifestyle and expression of the metabolic syndrome, as well as genetic factors [7–9].

Mortality risk in NAFLD is predicted by disease stage and is strongly influenced by the extent of liver fibrosis [10]. However, it should be noted that increased mortality in patients with NAFLD is strongly associated with non-hepatic comorbidities [11], with cardiovascular disease being a more common cause of death in NAFLD than liver-related complications. In this context, NAFLD has been identified as an independent risk factor for cardiovascular disease and cancer [12–14] as well as a predictor for the development of type 2 diabetes [6]. Extrahepatic comorbidities are thought to result from chronic low-grade inflammation in metabolically stressed patients ("metabolic inflammation"), some of which originates in the liver, so NAFLD may act as a possible cofactor and trigger of these underlying comorbidities [15]. Recently, there has also been increasing evidence of an association between NAFLD and the development and progression of chronic kidney disease (CKD) [16–18]. In this context, an updated and newly published large meta-analysis by Mantovani et al. showed a significantly increased long-term risk of developing CKD in patients with NAFLD [19]. CKD affects more than 25% of the population over the age of 65 [20]. Progression to end-stage renal disease (ESRD) is associated with high mortality. The need for renal replacement therapy or kidney transplantation contributes significantly to the individual burden of disease and socioeconomic costs [20]. Of note, the number of simultaneous liver and kidney transplants has increased exponentially in recent years. The risk factors and comorbidities of CKD are similar to those of NAFLD. In particular, CKD is associated with metabolic and vascular disease. The association between CKD and NAFLD may therefore be related to the high prevalence of both conditions in the general population and to common risk factors, or it may exist independently [16].

Given the enormous current and, more importantly, future burden on global healthcare systems due to the increasing number of simultaneous living kidney transplants and the costs related to the presence of CKD in the NAFLD population, this association could have far-reaching implications for the clinical care of NAFLD patients, making them a worthwhile target for screening and therapeutic intervention. This large database study was therefore designed to investigate the role of NAFLD in developing CKD, particularly in the German population.

## Materials and methods

### Database

The present study used data from the Disease Analyzer database (IQVIA). This database has already been extensively described in the literature [21]. To summarize, the Disease Analyzer database includes data on demographic variables, diagnoses, and prescriptions obtained in general and specialized practices in Germany. The quality of the data is assessed every month based on several criteria (e.g., completeness of documentation and linkage between diagnoses and prescriptions). Practices to include in the database are selected according to the yearly statistics of the German Medical Association, which include information on physician’s age, specialty group, community size category, and German federal state. Finally, it has been shown in prior research that the Disease Analyzer database is representative of all practices in Germany [21].

### Study population

**Fig. 1:**
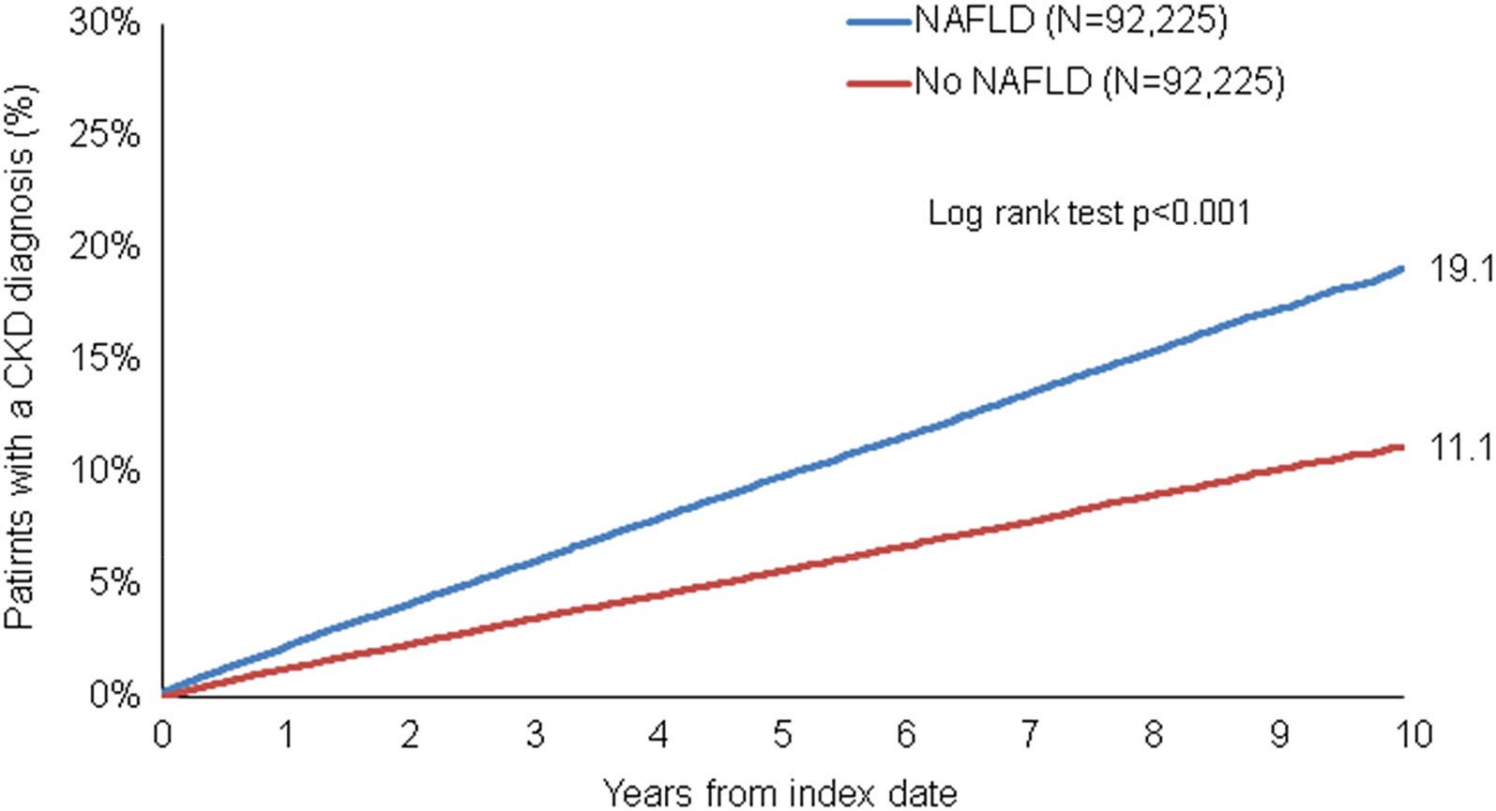
Kaplan–Meier curves for time to CKD diagnosis in patients with and without NAFLD

This retrospective cohort study included adult patients (≥ 18 years) with an initial diagnosis of NAFLD (ICD-10: K75.8, K76.0) in 1262 general practices in Germany between January 2005 and December 2020 (index date; Fig. 1). Further Inclusion criterium was an observation time of at least 12 months prior to the index date. Patients with other liver disorders (ICD-10: B18, K70-K77), renal failure (ICD-10: N18-N19), and diabetic renal complications (ICD-10: E10.2, E12.2, E13.2, E14.2) diagnoses prior to or on index date were excluded. NAFLD patients were matched to non-NAFLD individuals by propensity scores (nearest neighbor matching) based on sex, age, index year, yearly consultation frequency, hypertension (ICD-10: I10), and diabetes (ICD-10: E10-E14) diagnoses documented within 12 months prior to or at the index date (Additional file 1). As NAFLD patients have much higher consultation frequency by GPs, and higher consultation frequency can increase the probability of other diagnoses documentation, we included consultation frequency per year in the matching. Hypertension and diabetes were included as they are strongly associated with chronic kidney disease. For the non-NAFLD individuals, the index date was that of a randomly selected visit between January 2005 and December 2020.

### Study outcomes and statistical analyses

The main outcome of the study was the incidence of CKD (ICD 10: N18) as a function of NALFD.

Differences in the sample characteristics between those with and those without NAFLD were compared using the Wilcoxon signed-rank test for continuous age, the Stuart–Maxwell test for categorical age, and the McNemar test for sex and comorbidities. univariable Cox regression models were conducted to study the association between the NAFLD and CKD. These models were performed separately for women, men, and four age groups. Additionally, multivariable Cox regression models were constructed adjusted for prescriptions of insulin, diuretics, beta blockers, calcium channel blockers, ACE inhibitors, angiotensin II receptor blockers within 12 months prior to the index date. To counteract the problem of multiple comparisons, *p*-values < 0.01 were considered statistically significant. Analyses were carried out using SAS version 9.4 (SAS institute, Cary, USA).

## Results

### Characteristics of the study cohort

A total of 92,225 NAFLD patients as well as a propensity score matched cohort of 92,225 patients without NAFLD were included into analyses. The mean age was 57.4 years (SD: 14.2 years). 47.8% of patients were female. The mean yearly consultation frequency was 8.8 visits/year (SD: 6.5 visits/year). The prevalence of diabetes mellitus (27.1%) and arterial hypertension (56.3%) was well balanced between groups (*p* = 1.000). Table 1 provides a detailed overview of the cohort characteristics.

**Table 1:** Basic characteristics of the study sample after 1:1 matching

Variable	Proportion affected among patients with NAFLD (%) *N* = 92,225	Proportion affected among patients without NAFLD (%) *N* = 92,225	*p*-value
Age (mean, SD)	57.4 (14.2)	57.4 (14.3)	0.905
Age 18–50	30.4	30.4	0.826
Age 51–60	26.5	26.5	
Age 61–70	23.6	23.5	
Age > 70	19.5	19.6	
Women	47.8	47.8	1.000
Men	52.5	52.5	
Yearly consultation frequency (mean, SD)	8.8 (6.5)	8.8 (6.5)	1.000
Diabetes	27.1	27.1	1.000
Hypertension	56.3	56.3	1.000
Drug classes prescribed within 12 months prior to the index date			
Insulin	3.7	5.1	< 0.001
Diuretics	8.9	10.3	< 0.001
Beta blockers	20.4	22.8	< 0.001
Calcium channel blockers	10.4	10.9	0.002
ACE inhibitors	18.4	20.9	< 0.001
Angiotensin II receptor blockers	13.5	13.0	0.008

Proportions of patients in % given, unless otherwise indicated

*SD* standard deviation

### NAFLD is associated with an increased incidence of CKD

Over the 10-year study period, 19.1% of patients with NAFLD but only 11.1% of patients without NAFLD were newly diagnosed with CKD (Fig. 1, *p* < 0.001). Cox regression analysis confirmed the significant association between NAFLD and a subsequent diagnosis of CKD (hazard ratio (HR): 1.80, 95%CI: 1.73–1.86). Regarding the different age groups, this association was most pronounced in patients aged 18–50 years (HR: 2.13, 95%CI: 1.91–2.37, *p* < 0.001) and decreased slightly with increasing age (Table 2). The risk of developing CKD was numerically slightly higher in female NAFLD patients (HR female: 1.85, 95%CI: 1.76–1.95, *p* < 0.001 vs. HR male: 1.74, 95%CI: 1.66–1.83, *p* < 0.001, Table 2). Results of multivariable Cox regression models were similar to the univariate regression results.

**Table 2:** Association between NAFLD and the incident CKD diagnoses in patients followed in general practices in Germany (Cox regression models)

Cohort	Univariable Cox regression		Multivariable Cox regression^a^	
Cohort	Hazard ratio (95% CI)	*p* value	Hazard ratio (95% CI)	*p* value
Total	1.80 (1.73–1.86)	< 0.001	1.90 (1.83–1.97)	< 0.001
Age 18–50	2.13 (1.91–2.37)	< 0.001	2.20 (1.98–2.46)	< 0.001
Age 51–60	1.88 (1.73–2.05)	< 0.001	1.98 (1.82–2.15)	< 0.001
Age 61–70	1.73 (1.62–1.85)	< 0.001	1.82 (1.70–1.94)	< 0.001
Age > 70	1.66 (1.56–1.95)	< 0.001	1.72 (1.63–1.83)	< 0.001
Women	1.85 (1.76–1.95)	< 0.001	1.93 (1.83–2.03)	< 0.001
Men	1.74 (1.66–1.83)	< 0.001	1.87 (1.78–1.97)	< 0.001

^a^Multivariable Cox regression adjusted for prescriptions of insulin, diuretics, beta blockers, calcium channel blockers, ACE inhibitors, and angiotensin II receptor blockers

## Discussion

In this retrospective study, a large real-world cohort of more than 90,000 adult patients with NAFLD in Germany was compared with a 1:1 cohort of patients without NAFLD for incidence of CKD over a 10-year period using the Disease Analyzer database (IQVIA). Patients were matched for sex, age, index year, annual visit frequency, hypertension, and diabetes. Our results show that patients with NAFLD have a significantly increased risk of developing CKD. Within 10 years of the index date, 19.1% of patients with NAFLD were newly diagnosed with CKD, compared with only 11.1% of patients without NAFLD. This association was most pronounced in the 18- to 50-year age group.

Similar to our findings, a meta-analysis by Musso et al. with a total of 33 studies and over 63,902 participants showed that NAFLD is associated with an increased incidence and prevalence of CKD [22]. In addition, a retrospective study of 8,329 non-diabetic, non-hypertensive Korean men with normal renal function at baseline found that NAFLD was associated with an increased incidence of CKD after adjustment for age, cholesterol, and other factors over a 3-year period [23]. Another meta-analysis examined the magnitude of the association between NAFLD and the risk of developing CKD and comprised a total of 9 observational studies with a total of 96,595 adults of predominantly Asian descent and 4653 cases of stage ≥ 3 CKD over a median period of 5.2 years. The authors concluded that patients with NAFLD have a significantly higher risk of developing CKD than patients without NAFLD and that NAFLD is associated with a nearly 40 percent increased long-term risk of CKD [22]. A recently published updated large meta-analysis by Mantovani et al. of observational studies involving a total of 1,222,032 individuals (28.1% with NAFLD) and 33,840 cases of stage ≥ 3 CKD with a median follow-up of 9.7 years again confirmed that NAFLD is associated with a significantly increased risk of CKD. The authors demonstrated an approximately 1.45-fold increased long-term risk of developing stage ≥ 3 CKD in patients with NAFLD. All risks were independent of age, sex, obesity, hypertension, diabetes, and other common CKD risk factors [19].

Of note, in contrast to the study by Mantovani et al., which focused only on CKD ≥ 3 stages, our analysis included all 5 CKD stages as the primary end point because coding for each CKD subclass was not available. This fact may explain the higher risk of developing CKD in our study compared with the work of these authors (HR 1.8 vs 1.43). Furthermore, although the risk of CKD was numerically slightly increased in female NAFLD patients in our study, this observation could not be interpreted as a significant sex difference. While Mantovani et al. hypothesized that the observation of a sex-independent association was due to the fact that some of the underlying studies did not adequately adjust for sex differences, our data rather suggest that sex and most likely menopausal status are not effect modifiers associated with NAFLD and CKD [19].

The apparent decline in CKD incidence with increasing age demonstrated in our study (from 2.13 OR for 18–50 year olds to 1.66 (1.56–1.95) for > 70 year olds) also suggests that NAFLD adds to the risk of CKD at a time when traditional risk factors are less prominent.

Several pathophysiological mechanisms for the association between NAFLD and CKD have been discussed in the literature. Although our analysis cannot elucidate the mechanisms by which NAFLD contributes to the development of CKD, our study and others, including the recent meta-analysis by Mantovani et al., suggest that, far beyond its phenotype, NAFLD is a systemic disease whose pathophysiology and prognosis are determined by the involvement of multiple organ systems. The heterogeneity of effect sizes and incomplete penetrance imply that the pathogenetic pathways linking NAFLD to CKD are complex and determined by a variety of metabolic, genetic, epigenetic and dietary factors that are currently not fully understood and require further investigation [24, 25]. Recent evidence suggests that various metabolic processes in NAFLD may promote atherogenic dyslipidemia, induce hypertension, and trigger a chronic systemic inflammatory response leading to the development and progression of CKD [26].

There is increasing evidence of liver–kidney interactions in patients with NAFLD, including altered renin–angiotensin system (RAS) activation, impaired antioxidant defenses, and dysfunctional lipogenesis [27]. In this context, RAS may represent a possible link between NAFLD and CKD. It has been reported that RAS activation in the liver promotes insulin resistance, lipogenesis, and the production of proinflammatory cytokines such as interleukin-6 (IL-6) and tumor growth factor-β (TGF-β) [27, 28], which induces fibrogenesis and causes histological changes typical of NASH [27]. In the kidney, RAS activation plays a key role in the development of ectopic lipid deposition, which in turn leads to glomerulosclerosis through oxidative stress and inflammatory processes [29]. Understanding these mechanisms could help identifying therapeutic targets for the prevention and treatment of NAFLD and CKD. Furthermore, the role of the energy sensor 5′-AMP-activated protein kinase (AMPK) and its regulation of fetuin-A and adiponectin in liver and kidney cells was recently investigated [28, 30]. Fetuin-A is a serum protein mediated by AMPK as an important promoter of insulin resistance in both podocytes and hepatocytes [30] and has been found at elevated serum levels in patients with NAFLD and CKD [31]. Increased caloric intake and obesity are thought to trigger an inflammatory cascade between adipocytes in the liver and kidney via AMPK, fetuin-A and adiponectin, leading to end-organ damage [30].

The rapidly increasing prevalence of adipositas, type 2 diabetes and metabolic syndrome is a major challenge for the healthcare system. Currently, there is no approved drug therapy for NAFLD. The cornerstones of NAFLD treatment are lifestyle interventions (e.g., physical activity, weight reduction, diet modification) and control of metabolic syndrome and cardiovascular risk factors [32]. Based on the presumed pathophysiological mechanisms, several pharmacotherapeutic interventions are under investigation for the treatment of NAFLD. Although very few studies have examined the use of medications and behavioral modifications in both NAFLD and CKD, the common cardiometabolic risk factors and underlying pathophysiology may suggest that these therapies are applicable to both conditions.

Limited data suggest that RAS blockade with angiotensin receptor blockers (ARBs) reduces insulin resistance and inflammatory markers in patients with NAFLD steatosis independent of blood pressure lowering [27]. In addition, reductions in necroinflammation, NAFLD activity score, NASH fibrosis stage, and microalbuminuria were observed [33]. A recent cross-sectional study also showed that CKD-NAFLD patients taking angiotensin-converting enzyme inhibitors (ACE-I) or ARBs had less liver stiffness than patients not taking any medication [34]. Other research suggests that insulin-sensitizing agents, including thiazolidinediones (TZDs) such as pioglitazone, may be beneficial in the treatment of NAFLD [30].

We acknowledge that our study has some limitations that are mainly due to the study design and are therefore unavoidable. Because diagnoses were documented using ICD codes, we cannot exclude the possibility that incorrect or inadequate coding could lead to potential bias. Another limitation is that the analyses were not based on laboratory, imaging, or histologic findings, so the validity of the NAFLD codes in the Disease Analyzer database could not be confirmed. As a further limitation, the Disease Analyzer database does not provide more detailed information, such as laboratory data, liver or kidney histology, or clinical course, in addition to ICD-10 codes, which would have allowed more accurate stratification of NAFLD or more precise definition of CKD stage. The database also does not capture the mortality data, making it impossible to calculate survival of study patients. In addition, other risk factors for CKD, such as family history, smoking, or medication use, were not available for further analysis. The database contains electronic medical records from office-based physicians and no data from hospitals or dialysis centers. It should also be noted that the analyses are purely descriptive in nature, with only exploratory observations showing an independent association between CKD and NAFLD, but not proving a causal relationship.

However, the strengths of this work include the large number of patients included, the long study period of 10 years, and the use of a database whose representativeness and validity have already been proven [21].

## Conclusions

The present data confirm a significantly increased risk of developing CKD in a large real-world cohort of NAFLD patients in Germany and underscore that NAFLD should not be considered as an isolated liver disease, but rather as part of a systemic disease that requires structured diagnosis and follow-up as well as effective interventions. To address the individual risk profile of patients and their comorbidities, the care of patients with NAFLD should be multidisciplinary. We therefore suggest that the interdisciplinary care of patients with NAFLD, which is of increasing importance worldwide, should include systematic measures to prevent or screen for CKD to minimize long-term renal complications in this vulnerable patient population. However, to better understand the underlying pathophysiological mechanisms of the relationship between NAFLD and CKD, further studies are needed to provide information on liver and kidney histology and, in particular, to investigate inflammatory processes, oxidative stress, and fibrogenesis in the development of renal injury associated with fatty liver disease.

## Supplementary Information

**Additional file 1: Table S1** Standardized mean differences (SMD) prior and after propensity score matching.

## Data Availability

The datasets used and/or analyzed during the current study are available from the corresponding author on reasonable request.

## Abbreviations

ACE-I: Angiotensin-converting enzyme inhibitors
AMPK: 5′-AMP-activated protein kinase
ARBs: Angiotensin receptor blockers
CI: Confidence interval
CKD: Chronic kidney disease
ESRD: End-stage renal disease
HR: Hazard ratio
IL-6: Interleukin-6
NAFL: Non-alcoholic fatty liver
NAFLD: Non-alcoholic fatty liver disease
NASH: Non-alcoholic steatohepatitis
RAS: Renin–angiotensin system
SD: Standard deviation
TGF-β: Tumor growth factor-β
