# Supplementary material for: Non-alcoholic fatty liver disease (NAFLD) is associated with an increased incidence of chronic kidney disease (CKD)
Source: Eur J Med Res. 2023 Apr 17;28:153. doi: 10.1186/s40001-023-01114-6 (PMC10108448; doi:10.1186/s40001-023-01114-6)
Supplement: Supplementary file 1 — Additional file 1: Table S1 Standardized mean differences (SMD) prior and after propensity score matching. [file 40001_2023_1114_MOESM1_ESM.docx]

**Additional file 1**

**Table S1** Standardized mean differences (SMD) prior and after propensity score matching.

| Variable | SMD prior to matching  N=2,399,993 | SMD after matchng  N=92,225 |
| --- | --- | --- |
| Mean age | 0.42 | 0 |
| Age 18-50 (%) | -41.91 | -0.09 |
| Age 51-60 (%) | 22.55 | 0.05 |
| Age 61-70 (%) | 26.45 | 0.36 |
| Age >70 (%) | 1.82 | - 0.33 |
| Men (%) | 14.01 | 0 |
| Yearly consultation frequency | 0.34 | 0 |
| Diabetes (%) | 36.23 | 0 |
| Hypertension (%) | 48.72 | 0 |
|  |  |  |
